# Supplementary material for: Role of ruscogenin extracted from Radix Ophiopogon Japonicus in antagonizing 5-hydroxytryptamine and dopamine receptors through computational screening
Source: PLoS One. 2024 Nov 19;19(11):e0310960. doi: 10.1371/journal.pone.0310960 (PMC11575806; doi:10.1371/journal.pone.0310960)
Supplement: S1 Fig — (PDF) [file pone.0310960.s001.pdf]

Supporting information

Supporting information S1 Figs.

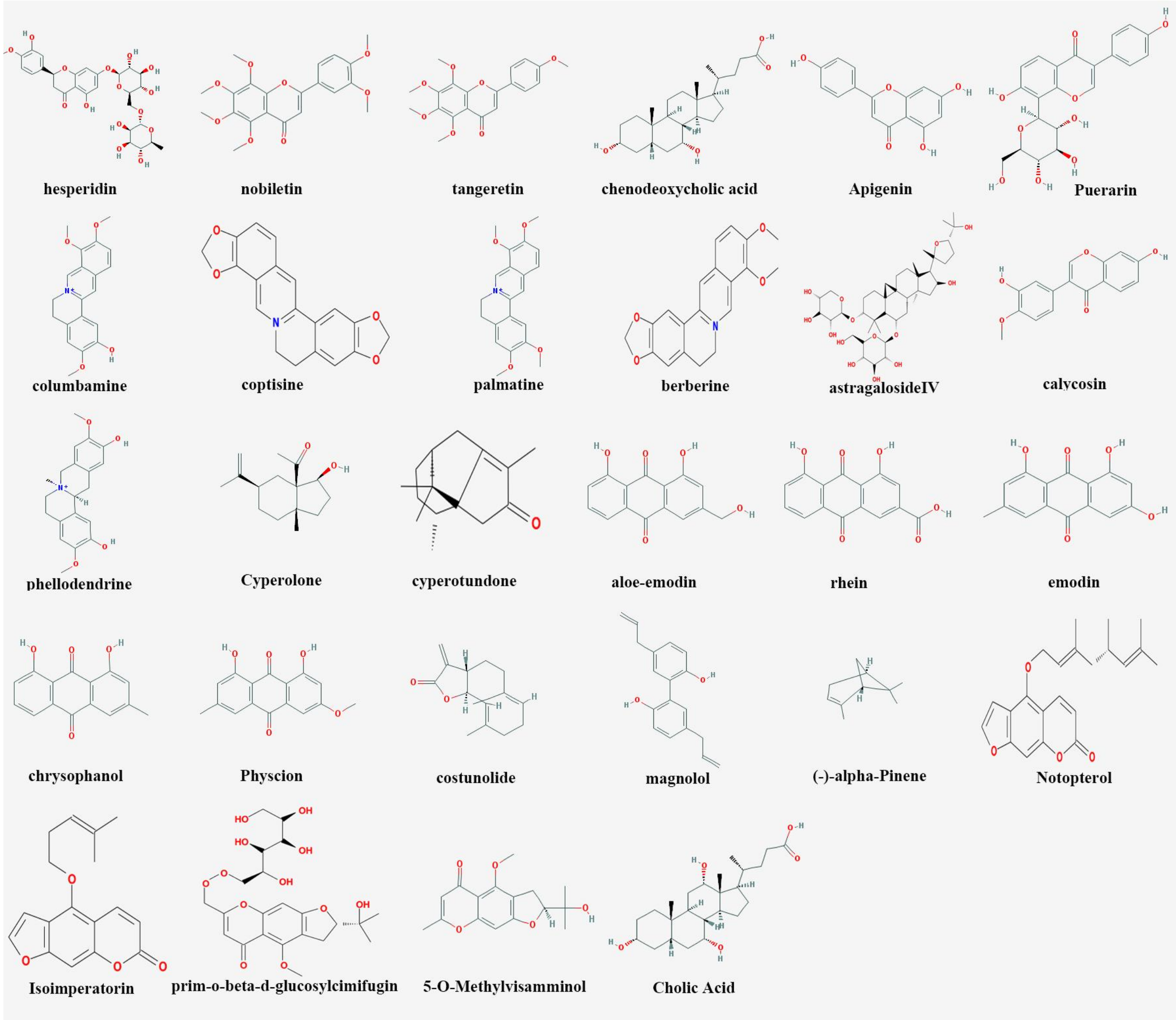

S1 Fig. 2D structure of natural product extracts.
