# Supplementary material for: Role of ruscogenin extracted from Radix Ophiopogon Japonicus in antagonizing 5-hydroxytryptamine and dopamine receptors through computational screening
Source: PLoS One. 2024 Nov 19;19(11):e0310960. doi: 10.1371/journal.pone.0310960 (PMC11575806; doi:10.1371/journal.pone.0310960)
Supplement: S1 File — Molecular docking results of natural product extracts with 5-HT2AR and DRD2. (PDF) [file pone.0310960.s002.pdf]

Supporting information

Supporting information S2-29 Figs.

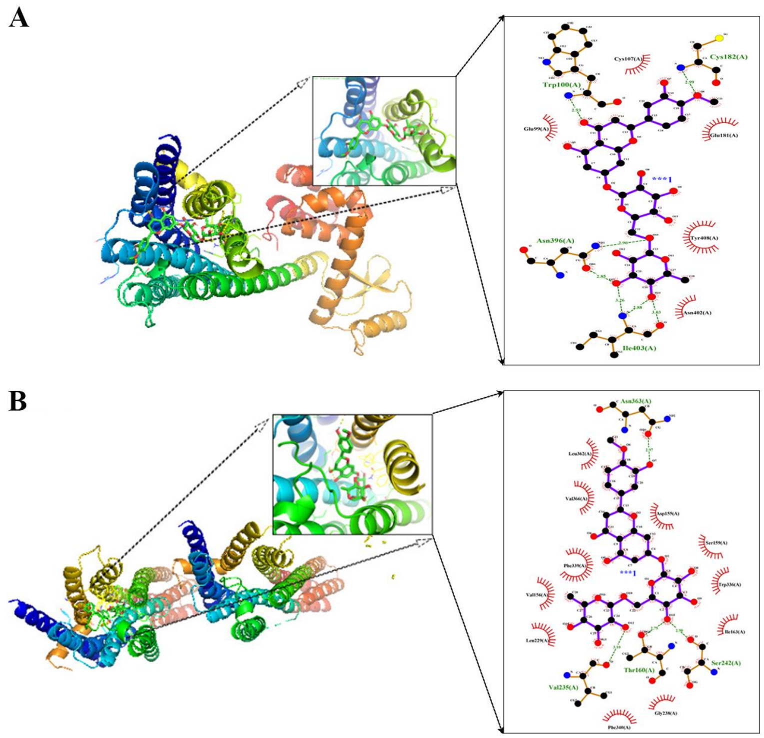

S2 Fig. The structure of hesperidin with DRD2 (A) and 5-HT2AR(B).

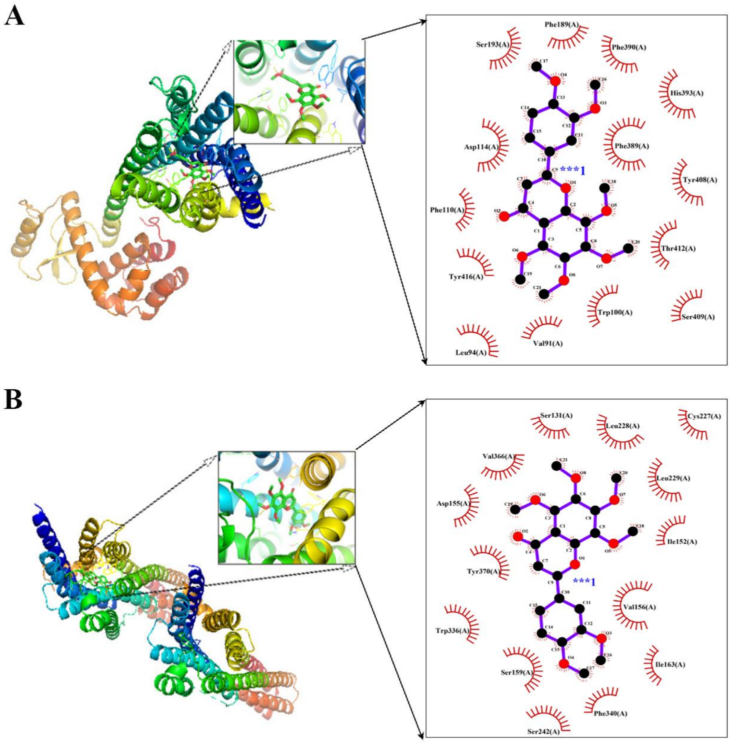

S3 Fig. The structure of nobiletin with DRD2 (A) and 5-HT2AR(B).

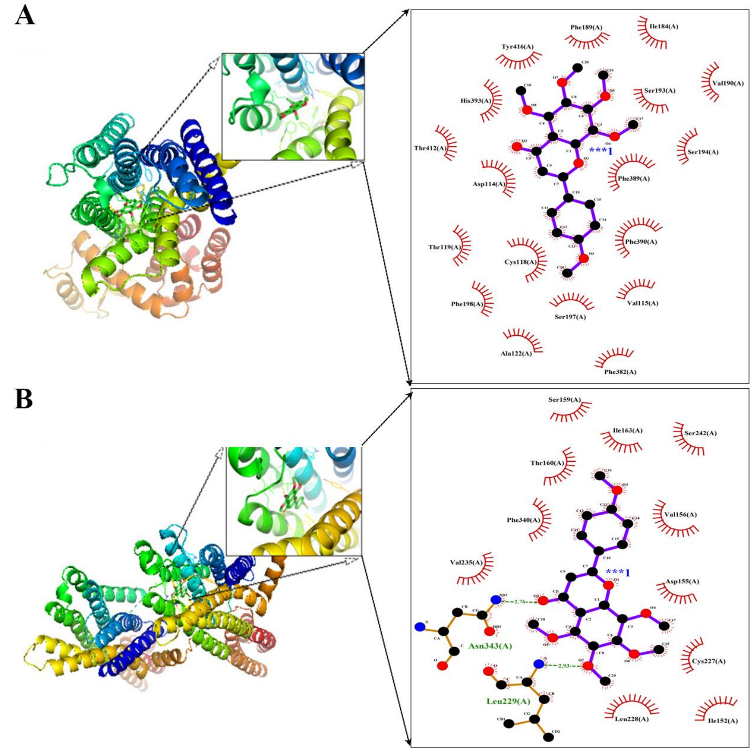

S4 Fig. The structure of tangeretin with DRD2 (A) and 5-HT2AR(B).

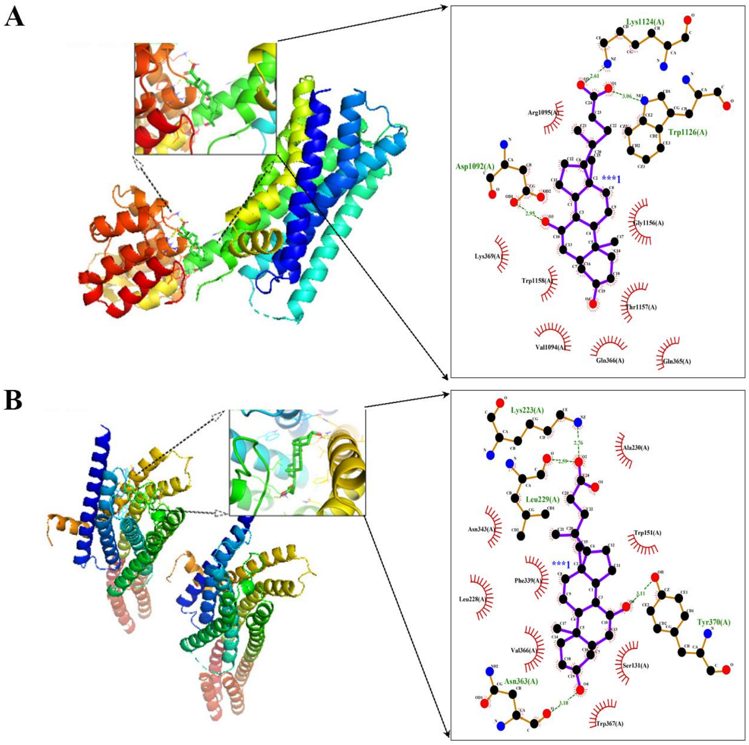

S5 Fig. The structure of chenodeoxycholic acid with DRD2 (A) and 5-HT2AR(B).

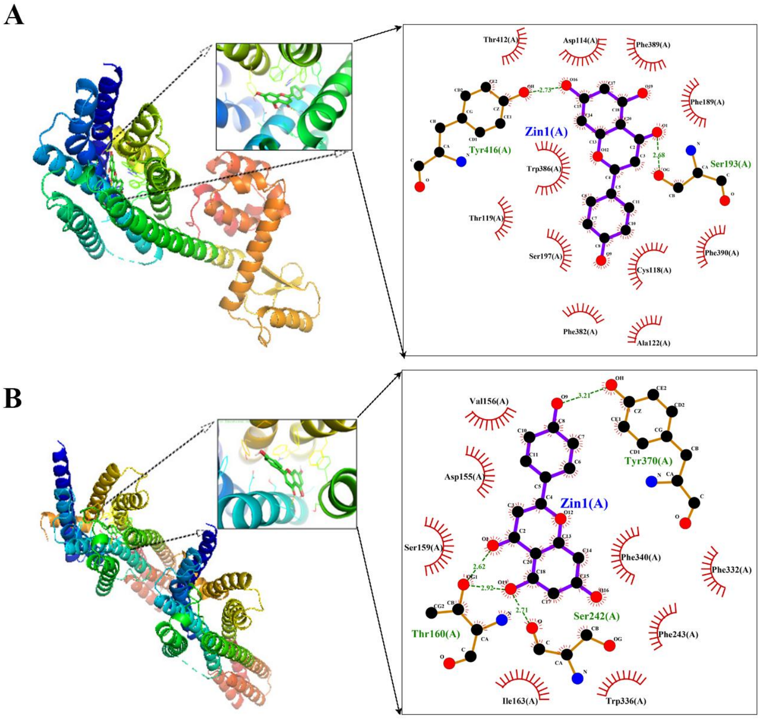

S6 Fig. The structure of Apigenin with DRD2 (A) and 5-HT2AR(B).

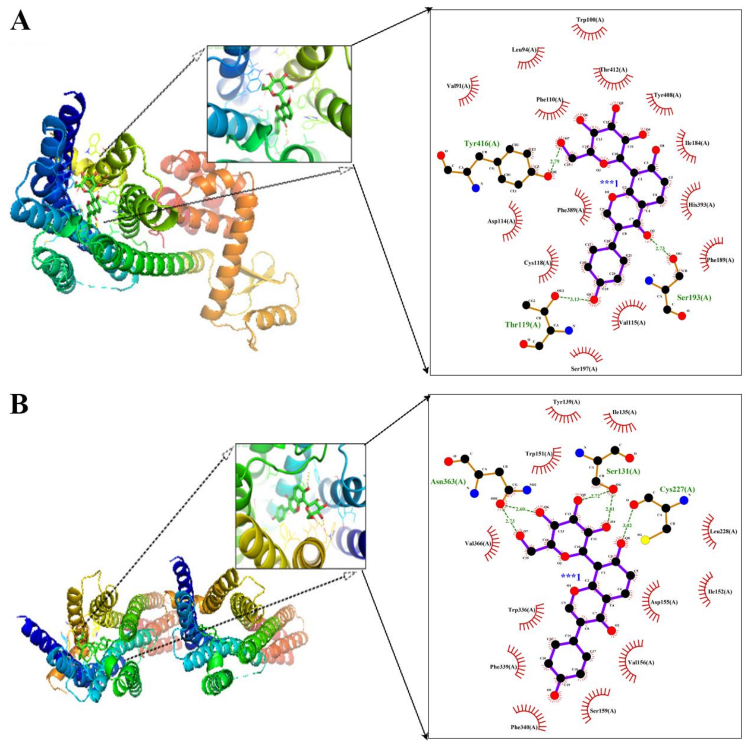

S7 Fig. The structure of Puerarin with DRD2 (A) and 5-HT2AR(B).

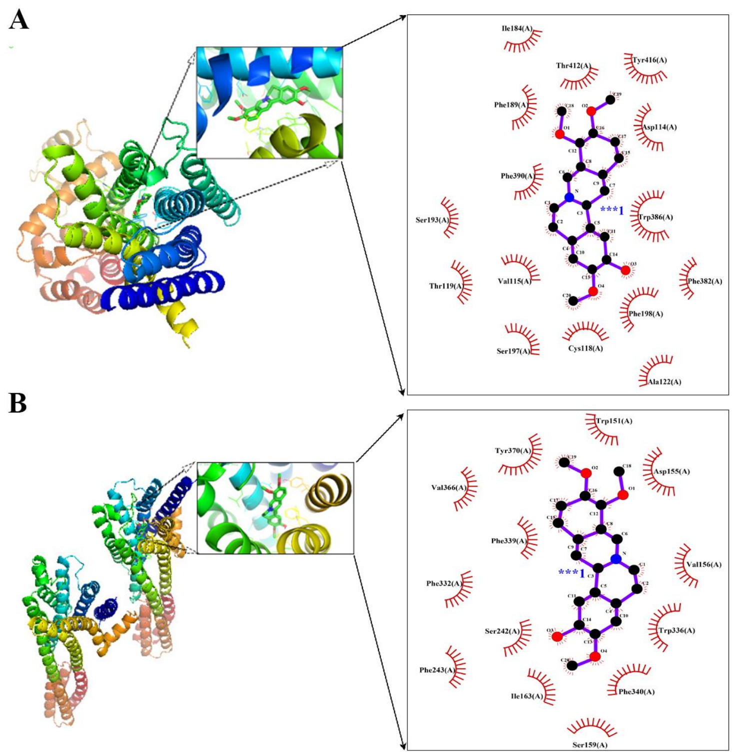

S8 Fig.The structure of columbamine with DRD2 (A) and 5-HT2AR(B).

A

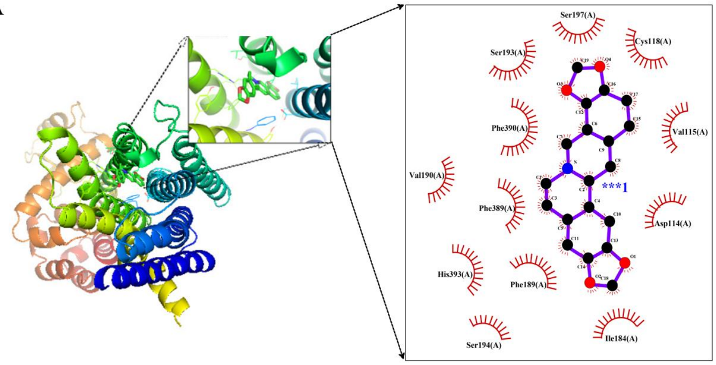

B

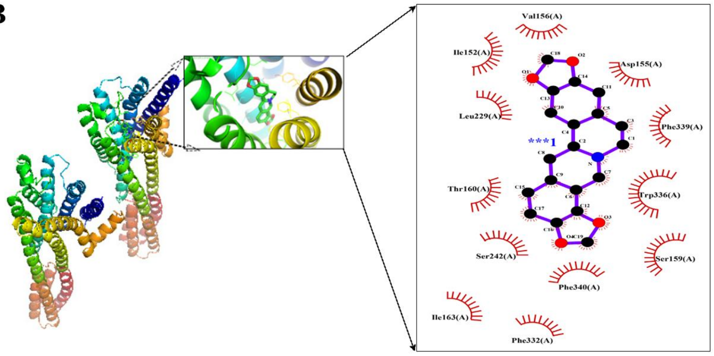

S9 Fig. The structure of coptisine with DRD2 (A) and 5-HT2AR(B).

A

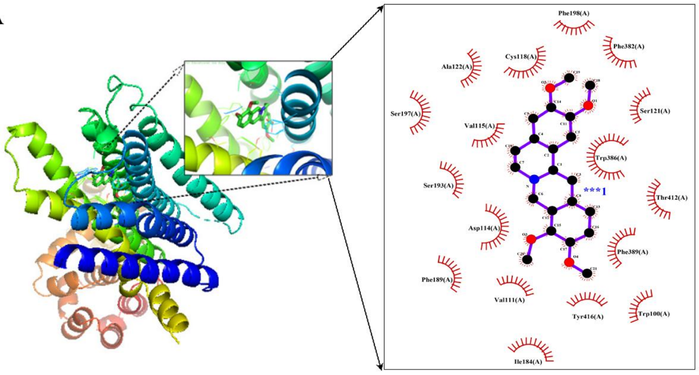

B

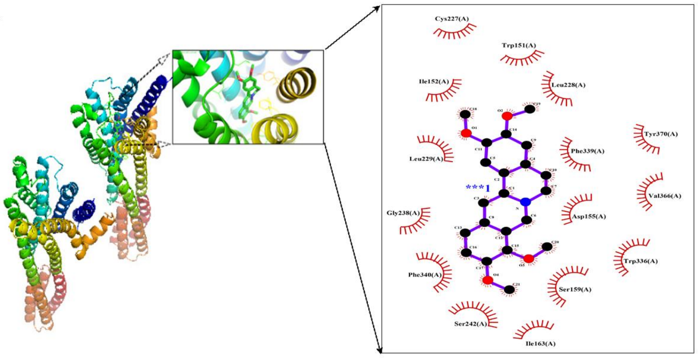

S10 Fig. The structure of palmatine with DRD2 (A) and 5-HT2AR(B).

A

B

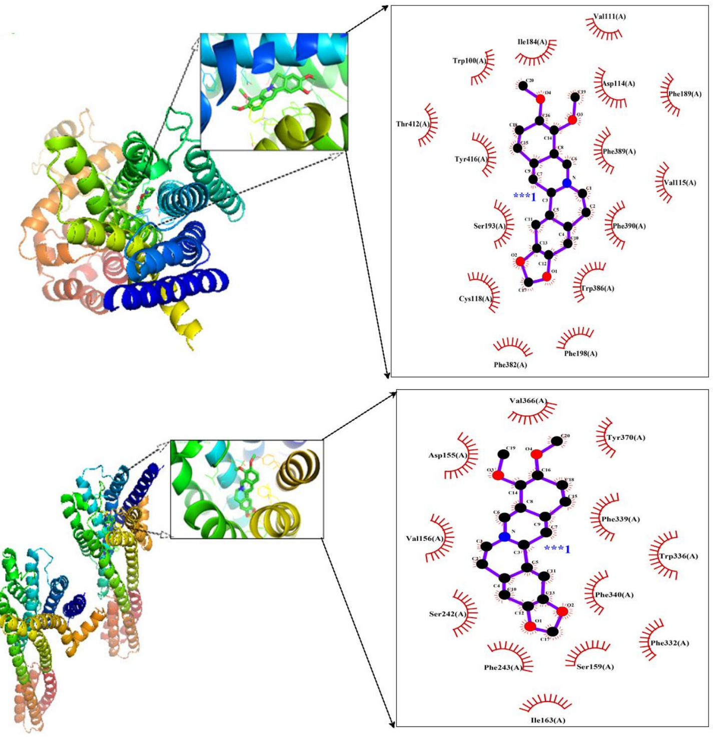

S11 Fig. The structure of berberine with DRD2 (A) and 5-HT2AR(B).

A

B

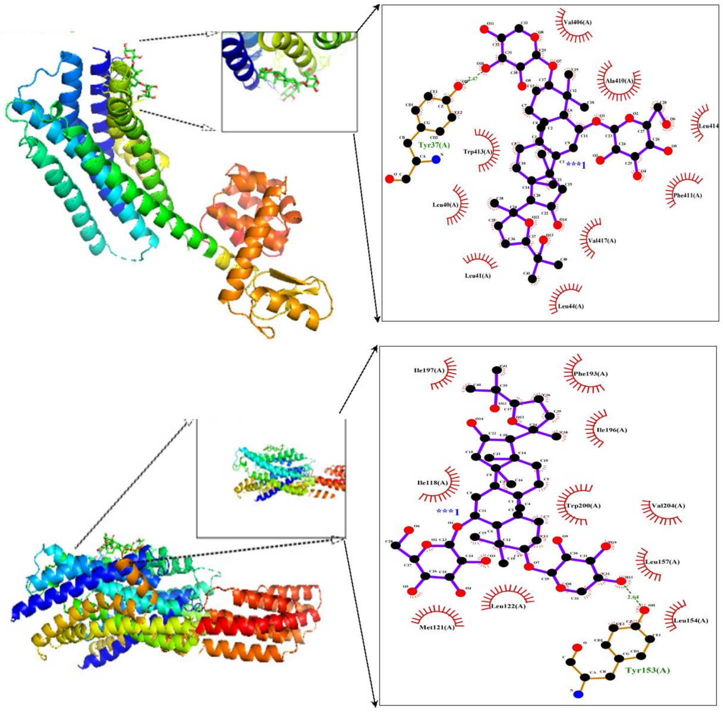

S12 Fig. The structure of astragalosideIV with DRD2 (A) and 5-HT2AR(B).

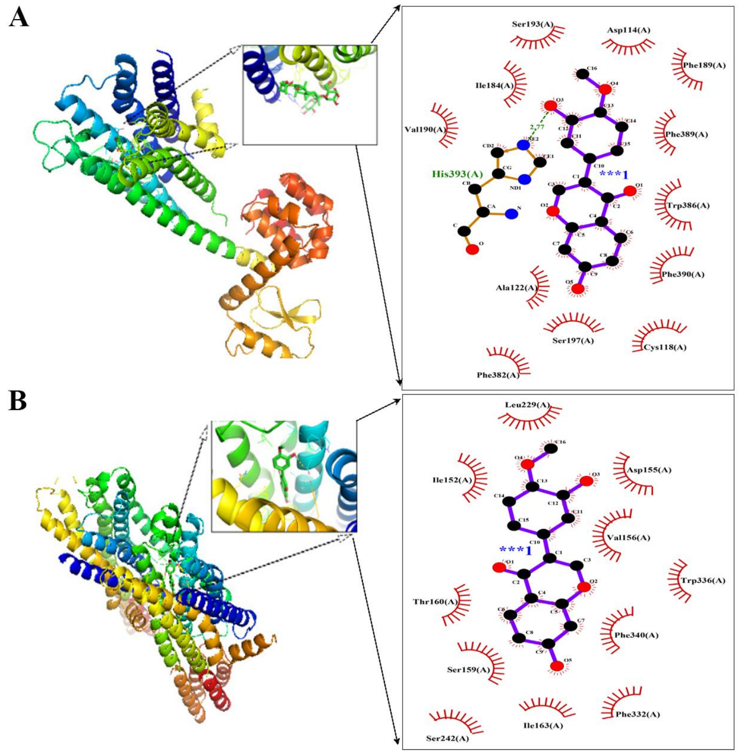

S13 Fig. The structure of calycosin with DRD2 (A) and 5-HT2AR(B).

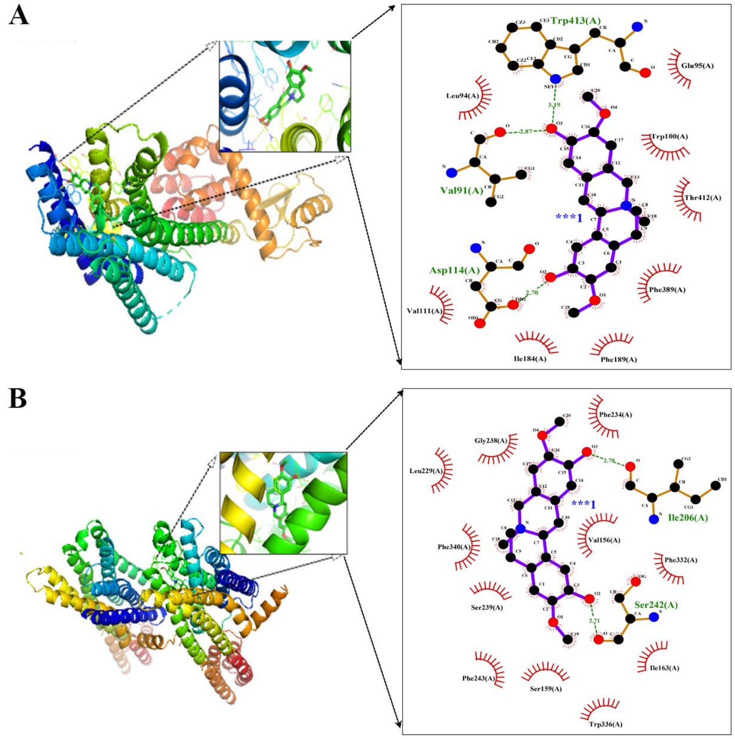

S14 Fig.The structure of phellodendrine with DRD2 (A) and 5-HT2AR(B).

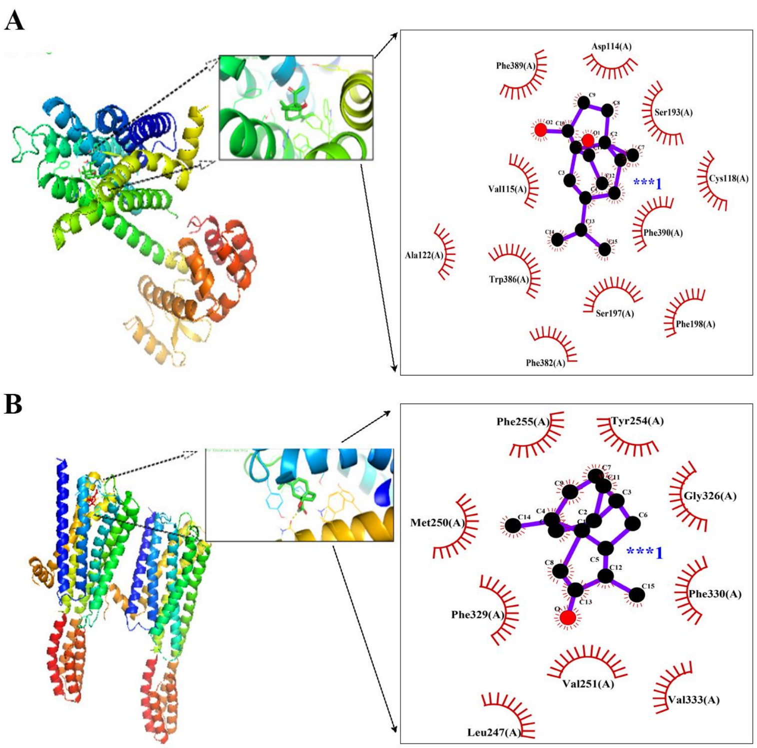

S15 Fig. The structure of Cyperolone with DRD2 (A) and 5-HT2AR(B).

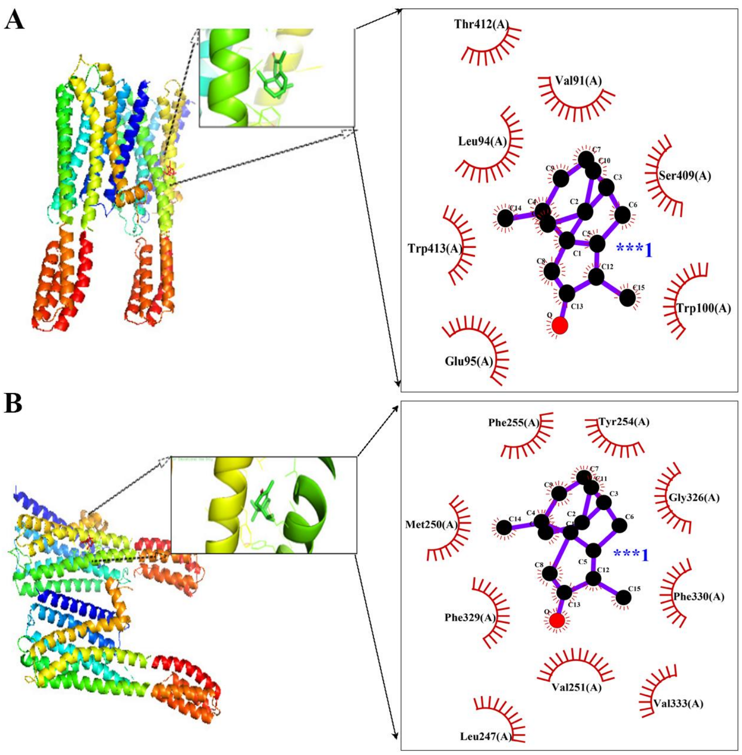

S16 Fig. The structure of cyperotundone with DRD2 (A) and 5-HT2AR(B).

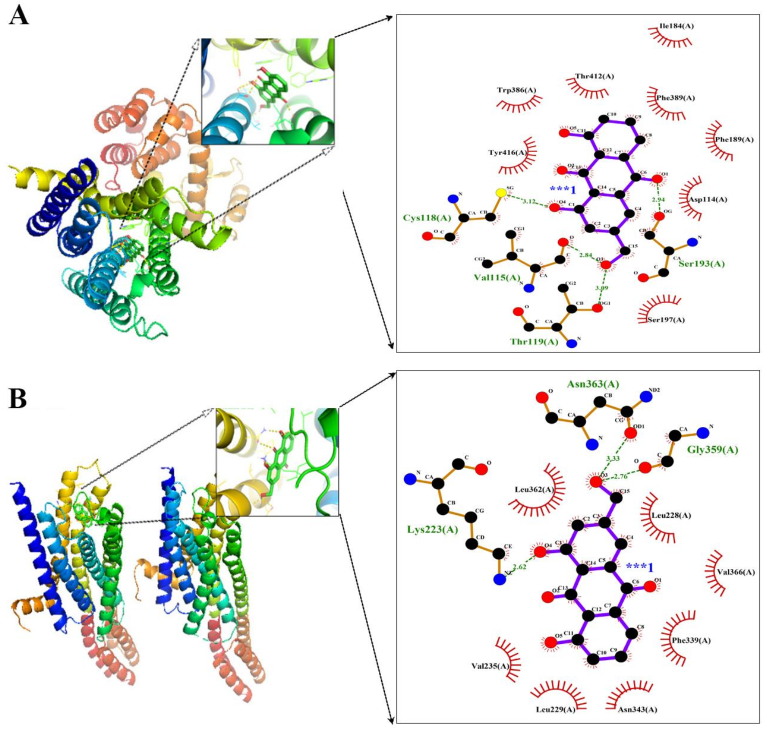

S17 Fig. The structure of aloe-emodin with DRD2 (A) and 5-HT2AR(B).

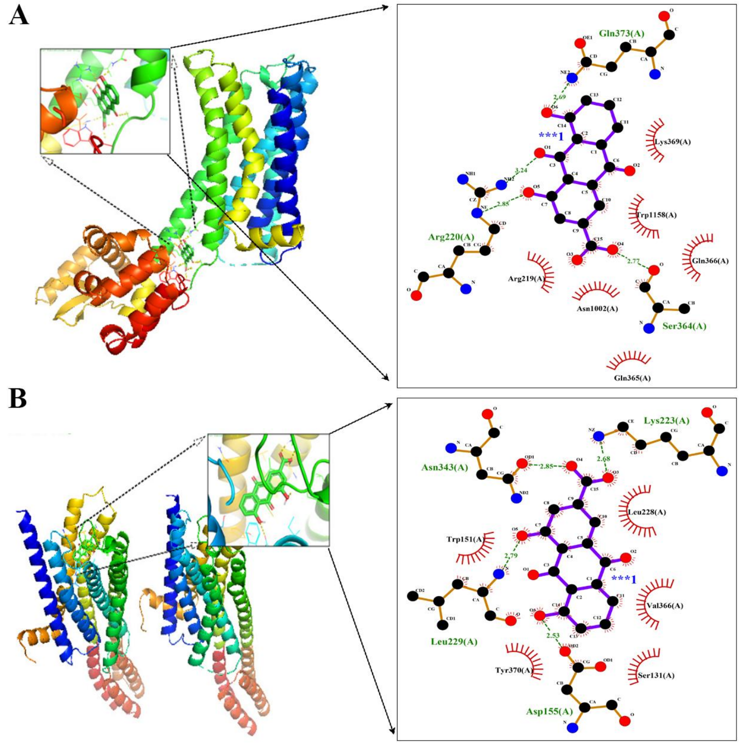

S18 Fig. The structure of rhein with DRD2 (A) and 5-HT2AR(B).

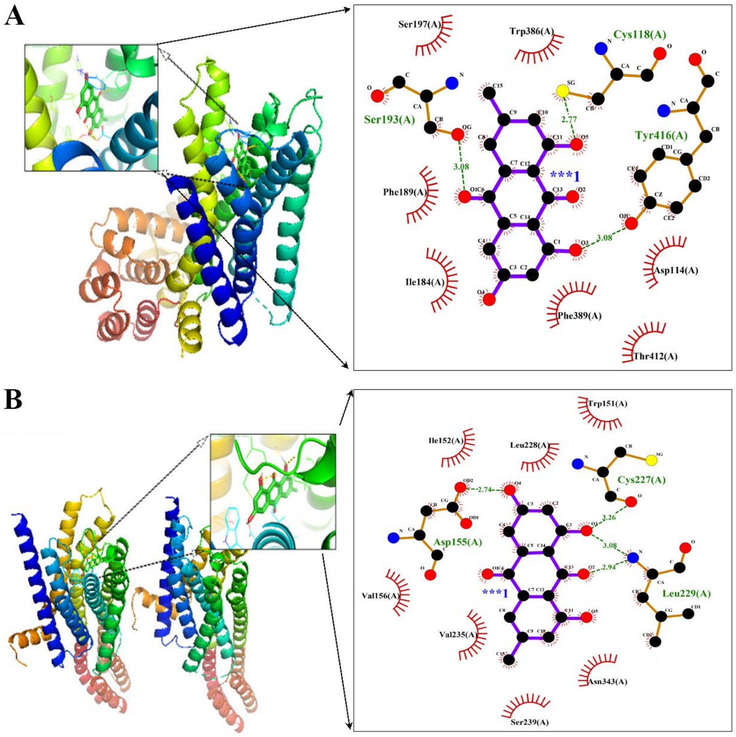

S19 Fig. The structure of emodin with DRD2 (A) and 5-HT2AR(B).

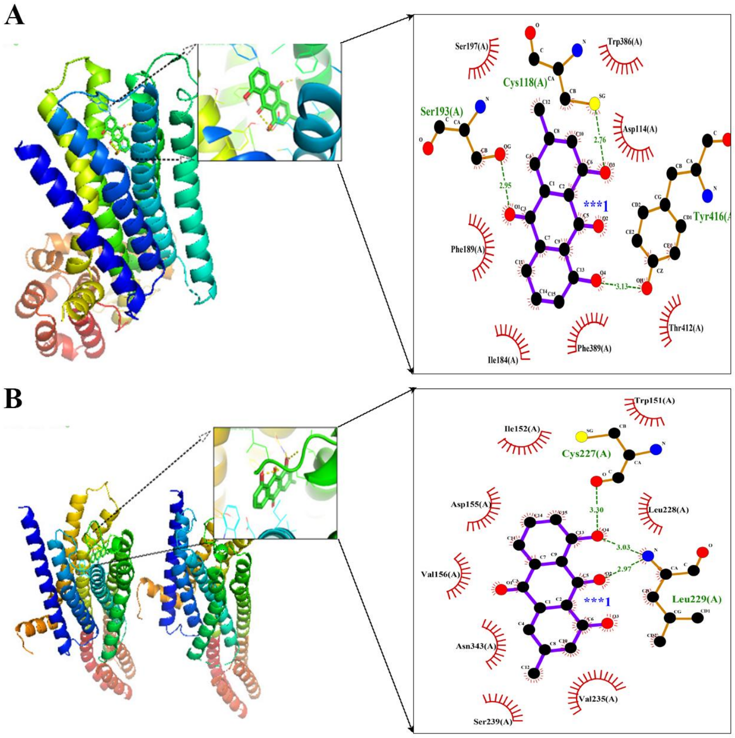

S20 Fig. The structure of chrysophanol with DRD2 (A) and 5-HT2AR(B).

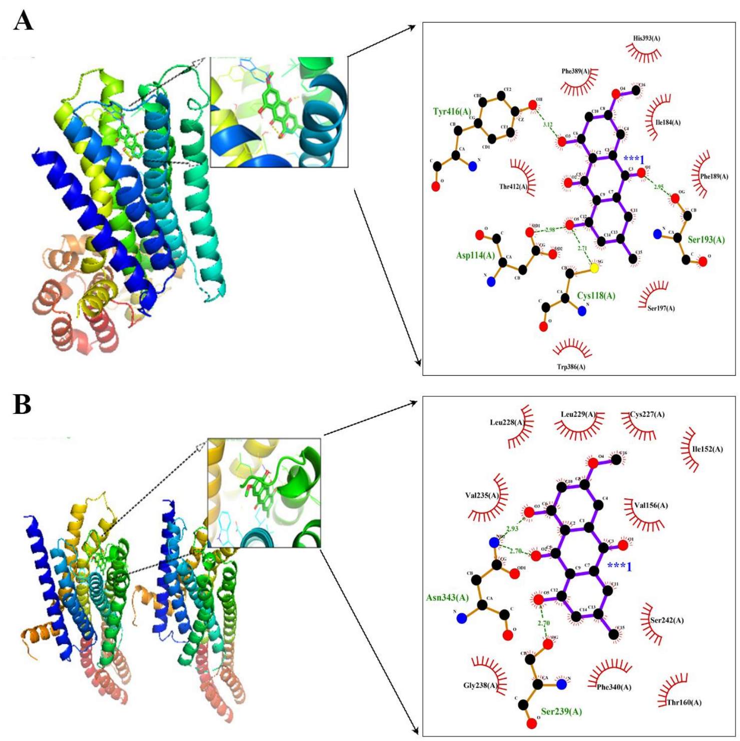

S21 Fig. The structure of Physcion with DRD2 (A) and 5-HT2AR(B).

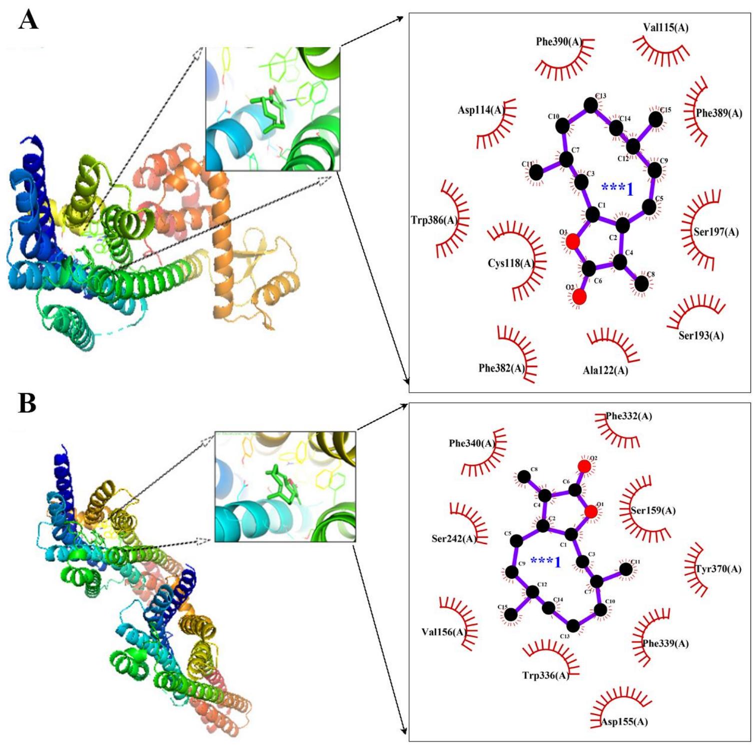

S22 Fig. The structure of costunolide with DRD2 (A) and 5-HT2AR(B).

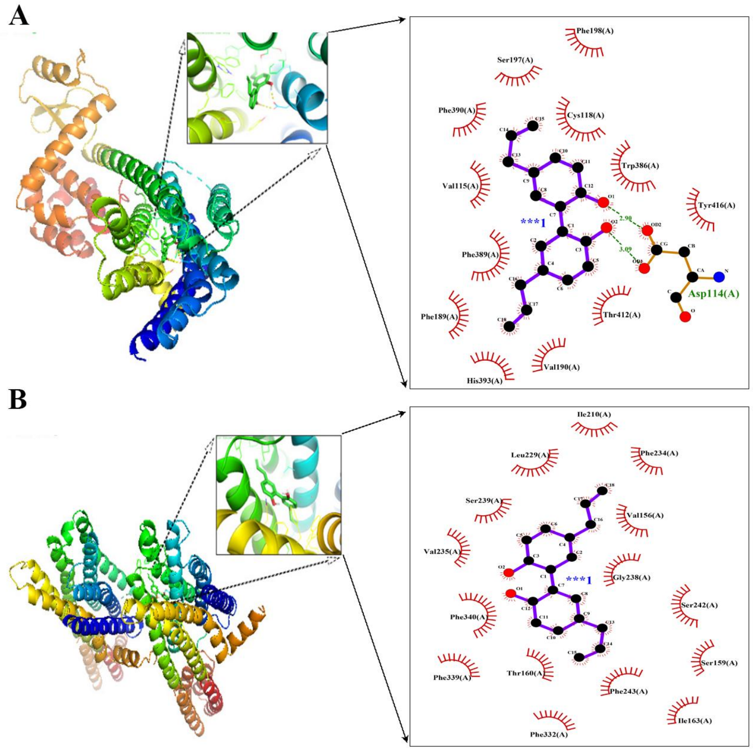

S23 Fig. The structure of magnolol with DRD2 (A) and 5-HT2AR(B).

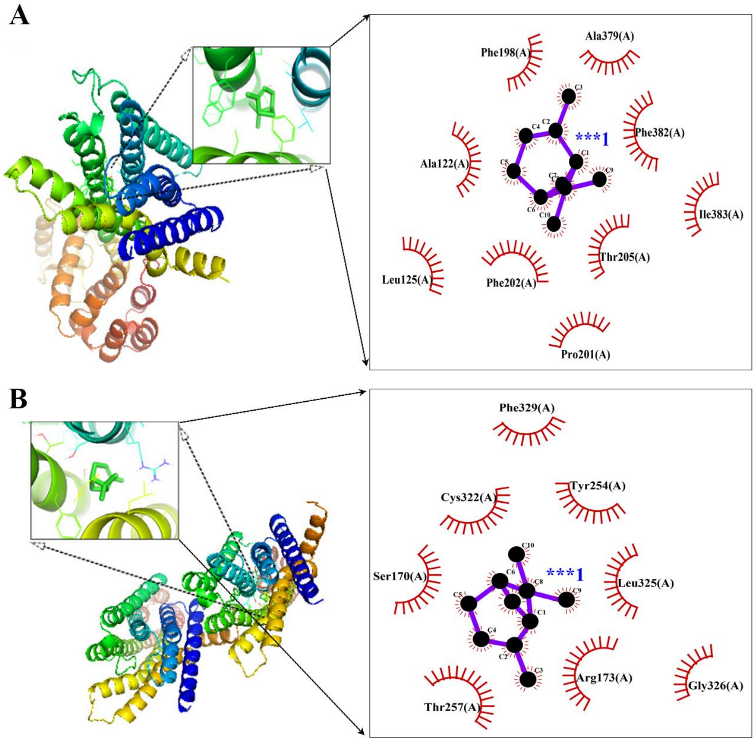

S24 Fig. The structure of (-)-alpha-Pinene with DRD2 (A) and 5-HT2AR(B).

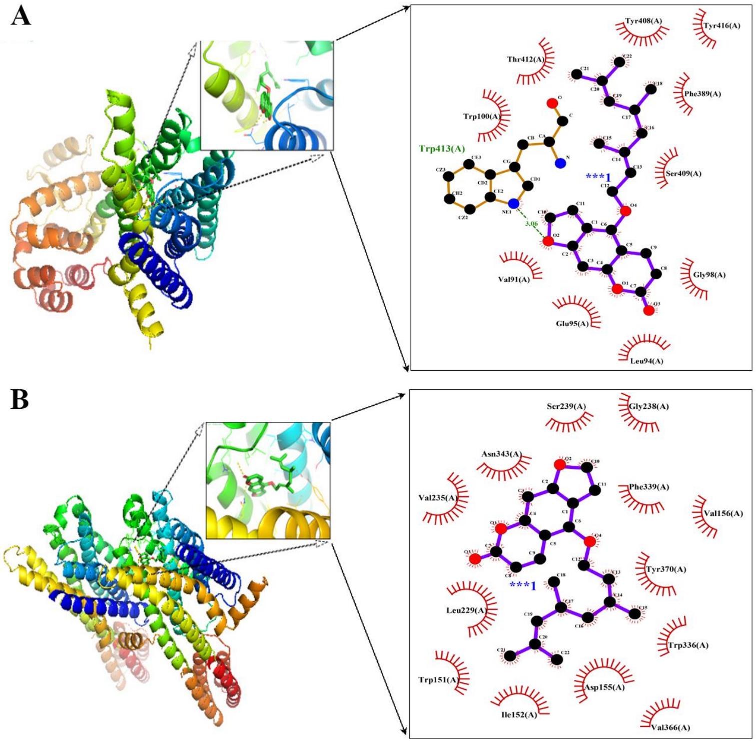

S25 Fig. The structure of Notopterol with DRD2 (A) and 5-HT2AR(B).

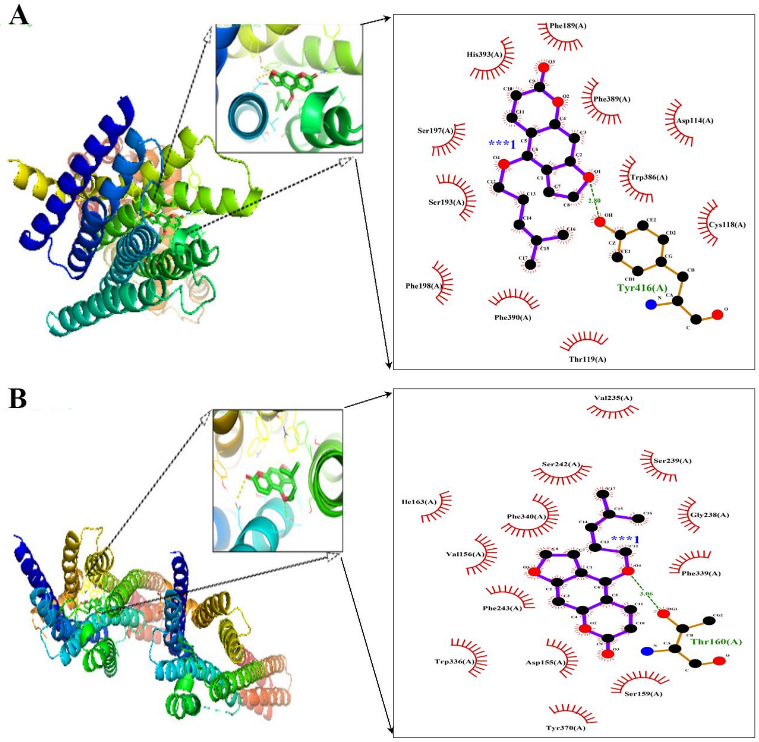

S26 Fig. The structure of Isoimperatorin with DRD2 (A) and 5-HT2AR(B).

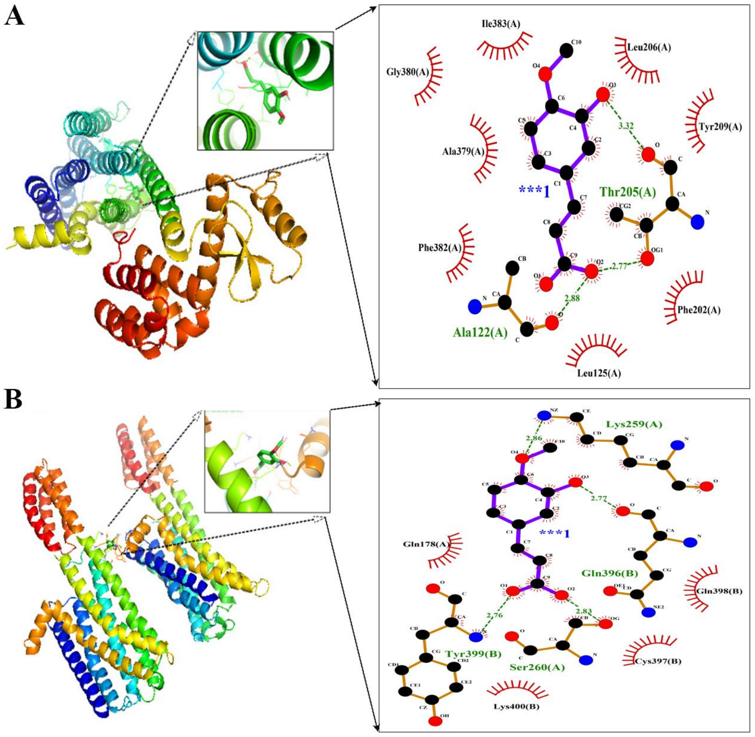

S27 Fig. The structure of prim-o-beta-d-glucosylcimifugin with DRD2 (A) and 5-HT2AR(B).

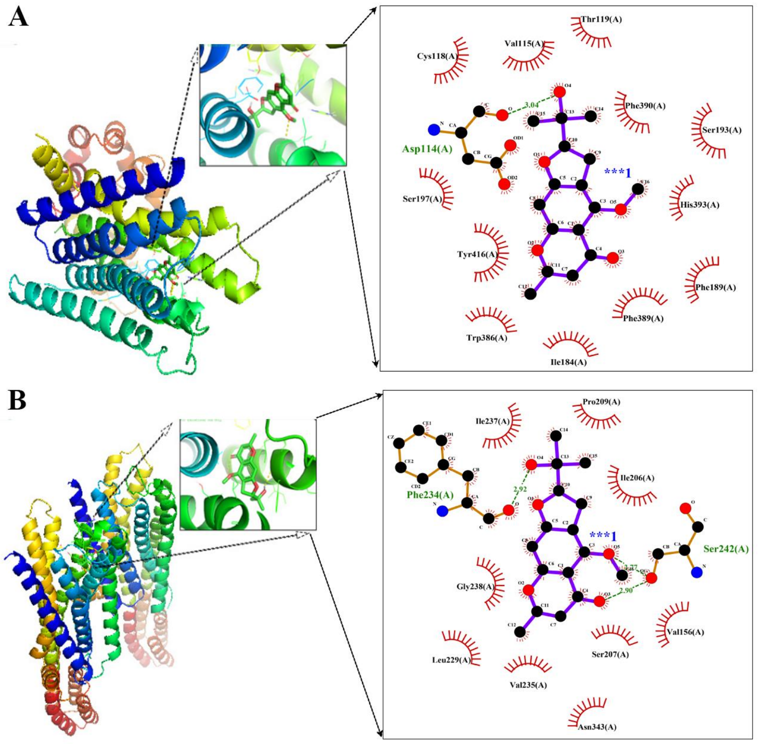

S28 Fig. The structure of 5-O-Methylvisamminol with DRD2 (A) and 5-HT2AR(B).

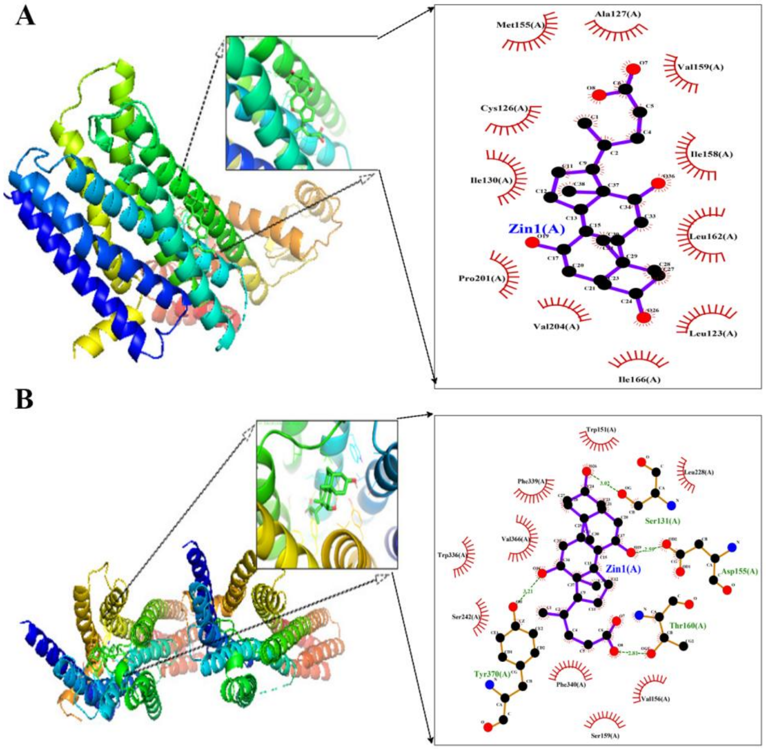

S29 Fig. The structure of Cholic Acid with DRD2 (A) and 5-HT2AR(B).
