# Supplementary material for: Role of ruscogenin extracted from Radix Ophiopogon Japonicus in antagonizing 5-hydroxytryptamine and dopamine receptors through computational screening
Source: PLoS One. 2024 Nov 19;19(11):e0310960. doi: 10.1371/journal.pone.0310960 (PMC11575806; doi:10.1371/journal.pone.0310960)
Supplement: S2 File — RMSD values for 5-HT2AR-chenodeoxycholic acid. (PDF) [file pone.0310960.s003.pdf]

Supporting information

Supporting information S30 Fig.

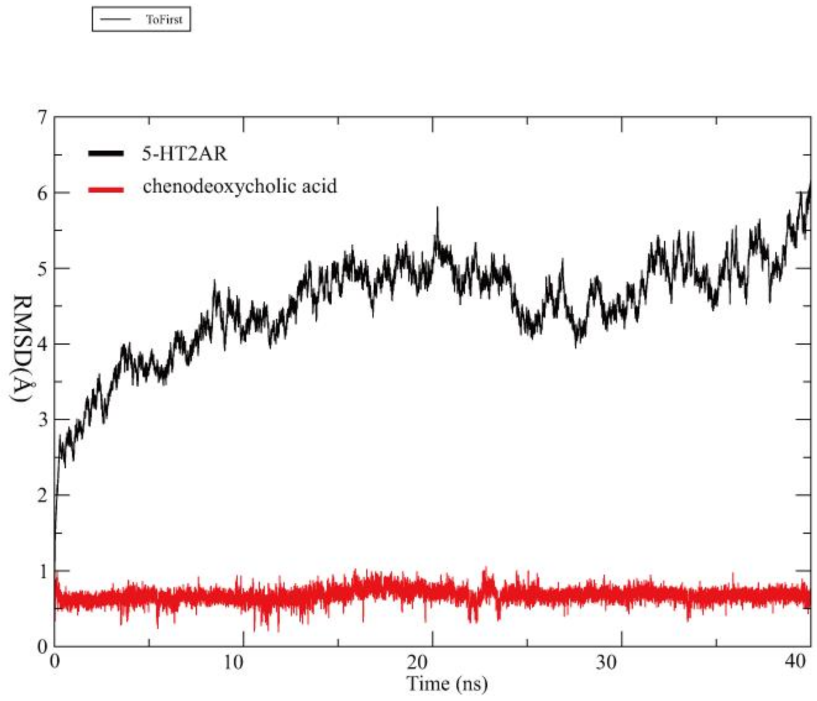

S30 Fig. RMSD values for 5-HT2AR-chenodeoxycholic acid.
