# Supplementary material for: Role of ruscogenin extracted from Radix Ophiopogon Japonicus in antagonizing 5-hydroxytryptamine and dopamine receptors through computational screening
Source: PLoS One. 2024 Nov 19;19(11):e0310960. doi: 10.1371/journal.pone.0310960 (PMC11575806; doi:10.1371/journal.pone.0310960)
Supplement: Attachment — Submitted filename: PONE-D-24-30011_review.docx [file pone.0310960.s004.docx]

**Review of**

**“Natural extracts in psychiatry: Ruscogenin's role in antagonizing 5-HT2A and DA2 receptors through computational screening”**

The search for effective and safe treatments for psychiatric disorders is an ongoing challenge in the field of medicine. Traditional antipsychotic drugs, although beneficial in managing symptoms, are often associated with significant adverse effects, including neurological complications, weight gain, and metabolic disruptions. These limitations underscore the urgent need for new therapeutic strategies that can offer improved efficacy and safety profiles. Natural products have long been recognized for their potential as sources of novel pharmaceuticals due to their diverse chemical structures and biological activities. Among these, ruscogenin (RUS), a steroidal sapogenin extracted from *Radix Ophiopogon japonicus*, has emerged as a candidate of interest. In this context, the submitted study aims to evaluate the potential of RUS as an effective inhibitor of the 5-HT2A and DA2 receptors, both of which are crucial in the pathology of psychiatric disorders such as schizophrenia and depression. Utilizing a combination of virtual molecular docking, ADMET analysis, and molecular dynamics (MD) simulations, the research seeks to explore the binding characteristics and drug-like properties of RUS compared to an established antipsychotic, risperidone.

While the study is promising and could potentially make a significant impact, several areas require revision to enhance the overall quality and clarity of the manuscript. Specifically, the following comments should be addressed:

**Title:**

- The title is long and contains multiple complex terms, which might make it difficult for readers to quickly grasp the main focus of the study.
- The title does not specify what RUS is, which can be confusing for readers unfamiliar with the term.
- Terms like "5-HT2AR" and "DRD2" are highly specific and may not be immediately understood: Technical Jargon

**Abstract:**

- Abstracts should summarize the key findings without overwhelming the reader with technical specifics.
- Some sentences are lengthy and complex, which can make the abstract harder to read.
- While the abstract mentions that RUS showed favourable properties and stability, it does not quantify these results or provide specific data points, which could help in understanding the magnitude of the findings.
- The conclusion suggests that RUS is a promising candidate for therapeutic development based on the in-silico results. However, it would be prudent to temper this with a statement about the need for further validation through in vitro and in vivo studies.

**Introduction:**

- The introduction covers a wide range of topics, from the general benefits of natural products to specific details about ruscogenin (RUS) and its potential therapeutic effects. While comprehensive, the flow can be confusing. A clearer structure with more defined sections would improve readability.
- The introduction is quite long and contains a lot of detailed information. While detail is important, some of the more specific data (chemical formulas, specific biological activities) might be better suited for later sections.
- Some sections, such as the detailed description of dopamine (DA) and serotonin (5-HT) receptors and their related pathologies, while informative, are quite extensive. This information might be better condensed to maintain focus on the main topic.
- The introduction lacks sufficient citations to support the claims made, especially in the initial paragraphs discussing the effectiveness of natural products and the biological activities of RUS.
- Transitions between different sections could be smoother.
- The introduction does not clearly state the objective or hypothesis of the study until the very end. Introducing the aim of the research earlier would provide context for the detailed information that follows.

**Methodology**

- The specific version of Autodock Vina should be clarified. If it is 1.2.0, ensure the correct reference is provided.
- Details on the parameters used for hydrogenation, charge calculation, and protonation should be more specific.
- The method for defining the binding pocket should be more explicitly described. Are specific residues or a particular region targeted?
- Explanation needed on why semi-flexible docking was chosen and how flexibility was introduced.
- Justification for selecting leaprc.protein.ff99SB and leaprc.gaff force fields. Are there specific advantages for this study?
- The rationale behind choosing a 12 Å gap and the TIP3P water model should be provided.
- Specify the concentration of Na+ or Cl- ions added.
- Explain why SHAKE is used specifically for hydrogen atoms.
- Explain why alanine scanning was chosen and how it helps in residue decomposition analysis.
- Provide clear explanations and justifications for each step and parameter choice to enhance reproducibility and understanding.

**Results:**

- The section mentions that "RUS was the best inhibitor," but it lacks details on the criteria used to determine this. Specify the binding affinity values or other metrics that led to this conclusion.
- There is a good level of detail regarding liposolubility, log P, log D7.4, solubility, and flexibility. However, the relationship between these properties and their practical implications for drug development should be more explicitly connected.
- The explanation of ADMET properties could benefit from more context regarding their relevance to drug development and specific thresholds that define "good" or "poor" properties.
- The results indicate stability but lack comparative data with controls or other compounds, which would help contextualize the stability of RUS and risperidone complexes.
- The explanation of RMSF results is clear, but more detail on the functional implications of the observed flexibility or rigidity in specific residues would be helpful.
- Relate the observed flexibility to the binding efficiency or stability of the protein-ligand complexes.
- The section provides a good description of the Rg values. However, it should explain the implications of high or low compactness on the function or interaction of the protein-ligand complexes.
- The comparative analysis between RUS and risperidone could be expanded to discuss why these differences in compactness matter.
- Include more precise language when describing scientific results, e.g., "indicating that it has good liposolubility and is easy to penetrate the cellular membrane structure" should be supported by numerical values and comparative benchmarks.
- Avoid vague statements.

**Discussion:**

- The discussion mentions that RUS showed the best inhibitory effect on 5-HT2AR and DRD2 but does not provide specific comparative metrics or data. Clear numerical results or statistical analysis would strengthen the claim.
- While the ADMET properties of RUS are discussed in detail, the practical implications of these properties in clinical settings are not fully explored. It would be beneficial to compare these properties more directly with those of existing drugs like risperidone to highlight the advantages and potential drawbacks.
- The discussion focuses on the binding properties and ADMET analysis but does not sufficiently address the clinical efficacy of RUS compared to risperidone.
- The discussion claims that RUS has a natural advantage in terms of safety but does not provide detailed safety data or potential side effects. Any adverse effects observed in preclinical or early clinical trials should be discussed. (if available)
- The discussion touches upon metabolic disruptions and weight gain associated with atypical antipsychotics but does not address whether RUS also carries these risks. Given the importance of side effects in the treatment of psychotic disorders, this aspect needs more attention.
- There is a gap in translating the molecular and computational findings into clinical relevance. How these findings might impact treatment protocols, patient outcomes, or the development of new therapies should be discussed.
- The discussion could benefit from a broader literature context. How do the findings about RUS fit within the current research landscape? Are there similar compounds being investigated? What are the next steps in the research and development process?

**Conclusion:**

- The conclusion repeats several points already mentioned in the discussion without adding new insights. Streamlining the conclusion to avoid redundancy would make it more concise and impactful.
- The conclusion mentions that RUS is a "potential therapeutic" but does not provide any context on the stages of clinical testing or the next steps required to bring this potential therapy to market. Discussing the clinical relevance and future directions would add depth. (in brief)
- The statement "RUS, a safe and effective natural extract" is an overgeneralization. The study should specify that safety and efficacy are inferred from preclinical analyses and that clinical trials are necessary to confirm these properties.
- The conclusion would benefit from a clearer structure. Presenting the findings in a logical sequence (starting with the discovery, moving to the detailed results, and ending with the broader implications and future directions) would improve readability.
- The conclusion lacks a connection to existing literature.

**References:** The paper should be enriched with more citations.

**Figures:** Authors should provide figures with higher resolution

**Supplementary files:** the intra-complexes interactions and bonds must be studied and represented (2D interaction visualization).
